# An investigation into the beneficial effects of high-dose interferon beta 1-a, compared to low-dose interferon beta 1-a (the base therapeutic regimen) in moderate to severe COVID-19: A structured summary of a study protocol for a randomized controlled l trial

**DOI:** 10.1186/s13063-020-04812-2

**Published:** 2020-10-26

**Authors:** Ilad Alavi Darazam, Firouze Hatami, Mohammad Mahdi Rabiei, Mohamad Amin Pourhoseingholi, Omid Moradi, Shervin Shokouhi, Mohammad Reza Hajesmaeili, Minoosh Shabani, Seyed Sina Naghibi Irvani

**Affiliations:** 1 Infectious Diseases and Tropical Medicine Research Center, Shahid Beheshti University of Medical Sciences, Tehran, Iran; 2 Department of Infectious Diseases, Loghman Hakim Hospital, Shahid Beheshti University of Medical Sciences, Tehran, Iran; 3 Gastroenterology and Liver Diseases Research Center, Research Institute for Gastroenterology and Liver Diseases, Shahid Beheshti University of Medical Sciences, Tehran, Iran; 4 Student Research Committee, Department of Clinical Pharmacy, School of Pharmacy, Shahid Beheshti University of Medical Sciences, Tehran, Iran; 5 Anesthesiology Research Center Loghman Hakim Hospital, Shahid Beheshti University of Medical Sciences, Tehran, Iran

**Keywords:** COVID-19, SARS-COV-2, Randomized controlled trial, lopinavir/ritonavir, Interferon-β 1a

## Abstract

**Objectives:**

We will investigate the effectiveness of high dose Interferon Beta 1a, compared to low dose Interferon Beta 1a (the base therapeutic regimen) in COVID-19 Confirmed Cases (Either RT-PCR or CT Scan Confirmed) with moderate to severe disease

**Trial Design:**

This is a single center, open label, randomized, controlled, 2-arm parallel group (1:1 ratio), clinical trial.

**Participants:**

The eligibility criteria in this study is: age ≥ 18 years, oxygen saturation (SPO2) ≤ 93% or respiratory rate ≥ 24, at least one of the following manifestation: radiation contactless body temperature ≥37.8, Cough, shortness of breath, nasal congestion/ discharge, myalgia/arthralgia, diarrhea/vomiting, headache or fatigue on admission. The onset of the symptoms should be acute (≤ 14 days). The exclusion criteria include refusal to participate, using drugs with potential interaction with lopinavir/ritonavir or interferon-β 1a, blood ALT/AST levels > 5 times the upper limit of normal on laboratory results, pregnant or lactating women, history of alcohol or drug addiction in the past 5 years, the patients who be intubated less than one hours after admission to hospital.

This study will be undertaken at the Loghman Hakim Hospital, Shahid Beheshti University of Medical Sciences.

**Intervention and Comparator:**

COVID- 19 confirmed patients (using the RT-PCR test or CT scan) will be randomly assigned to one of two groups. The intervention group (Arms1) will be treated with lopinavir / ritonavir (Kaletra) + high dose Interferon-β 1a (Recigen) and the control group will be treated with lopinavir / ritonavir (Kaletra) + low dose Interferon-β 1a (Recigen) (the base therapeutic regimen). Both groups will receive standard care consisting of the necessary oxygen support, non-invasive, or invasive mechanical ventilation.

**Main outcomes:**

Primary outcome: Time to clinical improvement is our primary outcome measure. This is an improvement of two points on a seven-category ordinal scale (recommended by the World Health Organization: Coronavirus disease (COVID-2019) R&D. Geneva: World Health Organization) or discharge from the hospital, whichever comes first.

Secondary outcomes: mortality from the date of randomization until the last day of the study which will be the day all of the patients have had at least one of the following outcomes: 1) Improvement of two points on a seven-category ordinal scale. 2) Discharge from the hospital 3) Death. Improvement of SPO2 during the hospitalization, duration of hospitalization from date of randomization until the date of hospital discharge or death, whichever comes first. The incidence of new mechanical ventilation uses from the date of randomization until the last day of the study and the duration of it will be extracted. Please note that we are trying to add further secondary outcomes and this section of the protocol is still evolving.

**Randomization:**

Eligible patients with confirmed SARS-Cov-2 infections will be randomly assigned in a 1:1 ratio to two therapeutic arms using permuted, block-randomization to balance the number of patients allocated to each group. The permuted block (three or six patients per block) randomization sequence will be generated, using Package ‘randomizeR’ in R software version 3.6.1. and placed in individual sealed and opaque envelopes by the statistician. The investigator will enroll the patients and only then open envelopes to assign patients to the different treatment groups. This method of allocation concealment will result in minimum selection and confounding biases.

**Blinding (Masking):**

The present research is open-label (no masking) of patients and health care professionals who are undertaking outcome assessment of the primary outcome - time to clinical improvement.

**Numbers to be randomised (sample size):**

Of the 100 patients randomised, 50 patients will be assigned to receive high dose Interferon beta-1a plus lopinavir/ritonavir (Kaletra), 50 patients will be assigned to receive low dose Interferon beta 1a plus lopinavir/ritonavir (Kaletra).

**Trial Status:**

Protocol version 1.2.1. Recruitment is finished, the start date of recruitment was on August 20^th^ 2020, and the end date was on September 4^th^ 2020. Last point of data collection will be the last day on which all of the 100 participants have had an outcome of clinical improvement or death, up to 14th days after hospitalization.

**Trial registration:**

This study was registered with National Institutes of Health Clinical trials (www.clinicaltrials.gov; identification number NCT04521400, https://clinicaltrials.gov/ct2/show/NCT04521400, registered August 18, 2020 and first available online August 20, 2020).

**Full protocol:**

The full protocol is attached as an additional file, accessible from the Trials website (Additional file 1). In the interest in expediting dissemination of this material, the familiar formatting has been eliminated; this Letter serves as a summary of the key elements of the full protocol.

**Supplementary information:**

**Supplementary information** accompanies this paper at 10.1186/s13063-020-04812-2.

## Supplementary information

**Additional file 1.**

## Data Availability

All the authors will have access to the final trial dataset. Furthermore, the data set the data will be available from the author on reasonable request (Contact: ilad13@yahoo.com)

