# Supplementary material for: An investigation into the beneficial effects of high-dose interferon beta 1-a, compared to low-dose interferon beta 1-a (the base therapeutic regimen) in moderate to severe COVID-19: A structured summary of a study protocol for a randomized controlled l trial
Source: Trials. 2020 Oct 26;21:880. doi: 10.1186/s13063-020-04812-2 (PMC7586665; doi:10.1186/s13063-020-04812-2)
Supplement: Supplementary file 1 — Additional file 1. [file 13063_2020_4812_MOESM1_ESM.docx]

**An Investigation into Beneficial Effects of High-dose Interferon Beta 1-a, Compared to Low-dose interferon Beta 1-a (the Base Therapeutic Regimen) in Moderate to Severe Covid-19: A Randomized Clinical Trial**

A single center, open label, randomized, controlled, parallel group, clinical trial

**Trial code**: NCT04521400

**Sponsored by:** Shahid Beheshti University of Medical Sciences

**Study phase:** Phase 2

**Trial Protocol Authors:**

Dr. Ilad Alavi Darazam, M.D

Dr. Firouze Hatami, M.D

Dr. Mohammad Mahdi Rabiei, M.D

Dr. Mohamad Amin Pourhoseingholi, PhD

Dr. Omid Moradi, Pharm. D

Dr. Shervin Shokouhi, M.D

Dr. Mohammad Reza Hajesmaeili, M.D

Dr. Minoosh Shabani, M.D

Dr. Seyed Sina Naghibi Irvani, M.D

**Coordinating Center:** Loghman Hakim Hospital

[**PROTOCOL SUMMARY** 3](#_Toc42265555)

[**INTRODUCTION** 5](#_Toc42265556)

[**Background** 5](#_Toc42265557)

[**STUDY OBJECTIVES** 7](#_Toc42265558)

[**Primary objective** 7](#_Toc42265559)

[**Secondary objectives** 7](#_Toc42265560)

[**Practical Objective** 8](#_Toc42265562)

[**Hypothesis** 8](#_Toc42265563)

[**STUDY DESIGN** 9](#_Toc42265564)

[**General** 9](#_Toc42265565)

[**Safety Assessments** 10](#_Toc42265566)

[**Efficacy Assessments** 10](#_Toc42265567)

[**Study Outcomes** 10](#_Toc42265568)

[**Study Drugs** 11](#_Toc42265569)

[**Statistical Methods** 11](#_Toc42265570)

[**STUDY POPULATION** 12](#_Toc42265571)

[**Participants** 12](#_Toc42265572)

[**Inclusion Criteria** 12](#_Toc42265573)

[**Exclusion Criteria** 12](#_Toc42265574)

[**Drop Out** 13](#_Toc42265575)

[**RESTRICTION** 13](#_Toc42265576)

[**ETHICAL CONCIDERATION** 14](#_Toc42265577)

[**REFERENCES** 15](#_Toc42265578)

**PROTOCOL SUMMARY**

**Study Title**: An Investigation into Beneficial Effects of High-dose Interferon Beta 1-a, Compared to Low-dose Interferon Beta 1-a (the Base Therapeutic Regimen) in Moderate to Severe Covid-19: A Randomized Clinical Trial

**Objectives:**

We will investigate the effectiveness of high dose Interferon Beta 1a, compared to low dose Interferon Beta 1a (the base therapeutic regimen) in COVID-19 Confirmed Cases (Either RT-PCR or CT Scan Confirmed) with moderate to severe disease.

**Trial Design:**

This is a single center, open label, randomized, controlled, parallel group, clinical trial that will be conducted at Loghman Hakim hospital in conjunction with Shahid Beheshti University of Medical Sciences.

**Participants:**

one hundred COVID-19 confirmed cases (using the RT-PCR test or CT scan) will be enrolled in the trial between August 20^th^ to September 4^th^, 2020. In this randomized controlled trial, patients will be assigned to the intervention group or the control group. The eligibility criteria in this study is: age ≥ 18 years, oxygen saturation (SPO2) ≤ 93% or respiratory rate ≥ 24, at least one of the following manifestation: radiation contactless body temperature ≥37.8, Cough, shortness of breath, nasal congestion/ discharge, myalgia/arthralgia, diarrhea/vomiting, headache or fatigue on admission. the onset of the symptoms should be acute (≤ 14 days).

The exclusion criteria include refusal to participate, using drugs with potential interaction with lopinavir/ritonavir or Interferon-β 1a, blood ALT/AST levels > 5 times the upper limit of normal on laboratory results, pregnant or lactating women, history of alcohol or drug addiction in the past 5 years, the patients who be intubated less than one hours after admission to hospital.

This study will be undertaken at the Loghman Hakim Hospital, Shahid Beheshti University of Medical Sciences.

**Intervention and Comparator:**

COVID- 19 confirmed patients will be randomly assigned to one of two group. The intervention group (Arms1) will be treated with lopinavir / ritonavir (Kaletra) + high dose Interferon-β 1a (Recigen) and the control group will be treated with lopinavir / ritonavir (Kaletra) + low dose Interferon-β 1a (Recigen) (the base therapeutic regimen).

**Efficacy Assessments**

**Primary outcome:**

Time to clinical improvement is our primary outcome measure. This is an improvement of two points on a seven-category ordinal scale (recommended by the World Health Organization: Coronavirus disease (COVID-2019) R&D. Geneva: World Health Organization) or discharge from the hospital, whichever comes first.

**Secondary outcomes:**

mortality from the date of randomization until the last day of the study which will be the day all of the patients have had at least one of the following outcomes: 1) Improvement of two points on a seven-category ordinal scale. 2) Discharge from the hospital 3) Death. Improvement of SPO2 during the hospitalization, duration of hospitalization from date of randomization until the date of hospital discharge or death, whichever comes first. The incidence of new mechanical ventilation uses from date of randomization until the last day of the study and the duration of it will be extracted. Please note that we are trying to add further secondary outcomes and this section of the protocol is still evolving.

**Statistical Methods:**

Statistical analysis will be performed by R version 3.6.1 software. We will use Kaplan–Meier to analyze the time to clinical improvement (compared with a log-rank test). Hazard ratios with 95% confidence intervals will be calculated using the Cox proportional-hazards model in crude and adjusted analysis.

**INTRODUCTION**

**Background**

Coronavirus 2019 (COVID-19) was declared as a pandemic by World health organization(Who) on March 11^th^, 2020. This disease has become of the major public challenge in the word[1].

This virus was named as severe acute respiratory syndrome coronavirus 2(SARS-COV-2) because genome analysis indicates that the new Coronavirus is the same subgenus as the SARS virus but in a different clade[2].

The receptor of COVID-19 binds to human angiotensin-converting enzyme (ACE -2) and entry to human cells[3, 4]. Pathophysiology studies indicate that occurs imbalance between inflammatory and anti-inflammatory cytokines[5]. cytokine storm results in multi-organ failure and death in COVID-19 pneumonia[6]. Therefore, it is worthwhile to use the immunomodulatory agents for the treatment of COVID-19 patients. Among immunomodulatory agents, Interferons(IFNs) have broad-spectrum effects on the immunes system including, antiviral, antiproliferative, developmental activates, and immunomodulatory[7].

In a clinical trial, the patients who were treated with IFN- β and antivirals lopinavir/ ritonavir had significantly shorter median time from the start of study treatment to negative nasopharynx swab compared to control group who were treated with antivirals lopinavir/ ritonavir[8].

Previous studies demonstrate that IFN-β 1a could be used against some coronaviruses including avium infectious, bronchitis virus, murine hepatitis virus, SARS- CoV because they are susceptible in vitro or in vivo[9].

In a current study, the efficacy of IFN-β 1a in COVID-19 patients was evaluated, and in this study, IFN-β 1a reduced the disease symptoms[10].

Because no effective treatment is available until now, intensive efforts are being made worldwide to identify and test promising antiviral drugs against COVID-19. Therefore, we performed a single-center, randomized, open-label, controlled trial to investigate the efficacy and safety of high-dose IFN-β 1a in combination with lopinavir/ritonavir compared with low-dose IFN-β 1a in combination with lopinavir/ritonavir (the base therapeutic regimen) in moderate to severe COVID-19 patients.

**STUDY OBJECTIVES**

**Primary objective:**

We will investigate the effectiveness of high dose IFN-β 1a compared to low dose IFN-β 1a (the base therapeutic regimen) in Confirmed Cases (Either RT-PCR and/or CT-scan Confirmed) with moderate to severe disease.

**The secondary objectives for this study are:**

1.To determine and compare the frequency of side effects in two therapeutic arms

2.To determine and compare the average level of oxygen saturation (Sat O2) in two therapeutic arms.

3.To determine and compare the average respiratory rate (RR) in two therapeutic arms

4.To determine and compare the average length of hospital stay in two therapeutic arms

5.To determine and compare the average length of hospital stay in the intensive care unit (ICU) between two therapeutic arms

6.To determine and compare the frequency of 30-days mortality rates between two therapeutic arms

7.To determine and compare the average time to improve clinical symptoms between two therapeutic arms

8.To determine and compare the average SOFA score in two therapeutic arms

9.To determine and compare the average duration of mechanical ventilation use between two therapeutic arms

**Practical Objective**

To introduce a new therapeutic regimen in order to reduce mortality rates and improve related symptoms in COVID-19 patients.

**Hypothesis**

• The frequency of side effects varies in each of the 2 treatment groups

• The average level of oxygen saturation (Sat O2) varies in each of the 2 treatment groups

• The average respiratory rate (RR) in each of the 2 treatment groups is different

• The average length of hospital stay varies in each of the 2 treatment groups

• The average length of hospital stay in ICU varies in each of the 2 treatment groups

• The frequency of 30-day mortality varies in each of the 2 treatment groups

• The average time until the nasopharyngeal swap test is negative in each of the 2 treatment groups is different

• The average time to clinical improvement in each of the 2 treatment groups is different

• The average SOFA score varies in each of the 2 treatment groups

• The average duration of mechanical ventilation varies in each of 2 treatment groups

**STUDY DESIGN**

**General**

This is a single center, open label, randomized, controlled, parallel group, clinical trial that will be conducted at Loghman Hakim Medical Education Center in conjunction with Shahid Beheshti University of Medical Sciences.

Eligible patients with confirmed SARS-Cov-2 infections will be randomly assigned in a 1:1ratio to one of the two following therapeutic regiments: 1) IFN-β1a (Recigen) (Subcutaneous injections of 88μg (24,000 IU) on days 1, 3, 6) + lopinavir/ritonavir (Kaletra) [IFN-β1b group], 2) IFN-β1a (Recigen) (Subcutaneous injections of 44μg (12,000 IU) on days 1, 3, 6) + lopinavir/ritonavir (Kaletra) [IFN-β1b group] (400mg/100 mg twice a day for 10 days, orally, in all two arms) [control group]. All two groups will receive standards of care consisting of the necessary oxygen support, non-invasive, or invasive mechanical ventilation.

Patients will be randomly allocated to two therapeutic arms using permuted, block-randomization to balance the number of patients allocated to each group. The permuted block (three or six patients per block) randomization sequence will be generated, using Package ‘randomizeR’ in R software version 3.6.1. and placed in individual sealed and opaque envelopes by the statistician. The investigator will enroll the patients and only then open envelopes to assign patients to the different treatment groups. This method of allocation concealment will result in minimum selection and confounding biases.

The present research is open-label (no masking) of patients and health care professionals who are undertaking outcome assessment of the primary outcome - time to clinical improvement.

**Safety Assessments**

Regarding safety concerns, daily monitoring for adverse effects (AEs) and treatment-related AEs, vital signs, and laboratory testing will be carried out. All adverse effects should be recorded and include time, severity, symptoms, and their relation with aforementioned drugs.

**Efficacy Assessments**

**Study outcomes**

**Primary outcome:**

Our primary outcome measure will be TTCI, defined as the time from enrollment to discharge from the hospital or a decline of two steps on the seven-step ordinal scale; Which so ever came first. Originally introduced by Beigel and colleagues in a posthoc analysis of an influenza study as a six-step ordinal scale, and currently recommended by the WHO R&D Blueprint Team (Accessed May 15, 2020, at https://www.who.int/teams/blueprint/covid-19) for COVID-19 studies as a nine- step ordinal scale, the utilized seven-step ordinal scale consists of the subsequent categories: (I) Not hospitalized, and has no activity limitations; (II) Not hospitalized, but has activity limitations; (III) Hospitalized, but does not need any supplemental oxygen; (IV) Hospitalized, and needs supplemental oxygen; (V) Hospitalized, and needs either High-Flow Nasal Cannula (HFNC) or non-invasive ventilation; (VI) Hospitalized, and needs invasive ventilation; and (VII) Dead.

**Secondary Outcome:**

Secondary outcomes include mortality from the date of randomization until day 21, by which all of the patients will have at least one of the following outcomes: 1) A decline of two steps on the seven-step ordinal scale, 2) Hospital discharge or 3) Death; SpO2 improvement defined as the difference between the last and the first recorded measurement during the hospitalization, using pulse-oximetry; length of stay in the hospital until the date of discharge from hospital or death from any cause, whichsoever came first; incidence of new mechanical ventilation uses from the date of randomization until day 21. Follow-ups of discharged patients will be done utilizing telemedicine visits, online, or over the telephone.

**Study Drugs**

**•lopinavir/ritonavir (Kaletra):**

400mg/100mg bid for 10-14 days

For those that could not get orally:

5-ml suspension bid for 10-14 days

•**Interferon beta-1a:**

In intervention group: SC injection 88 micro-gram for 3 days (on days 1,3,6)

In control group: SC injection 44 micro-gram for 3 days (on days 1,3,6)

**Statistical Methods**

Statistical analysis will be performed by R version 3.6.1 software. The total sample size was calculated according to the Latouche and colleagues approach for estimating sample size in survival analysis with 80% power, alpha=0.05, Hazard Ratio (HR) of 3.0 (as the ratio of the hazard rates of time to clinical improvement (TTCI) corresponding to the intervention group compared to the control group) and assuming that 80% of patients would reach the primary outcome[11]. The calculations will be carried out using Package ‘powerSurvEpi’ in R and accounted for a dropout rate of 15%. Overall 100 patients, will be needed for the study, 50 for each arm.

Frequency rates and percentages will be used for categorical variables, and Interquartile Ranges (IQRs) and median will be used for continuous variables. Kruskal-Wallis test will be used for comparing the continuous variables. The Wilcoxon signed-rank test will compare the before and after intervention effects. Moreover, Chi-Square test will compare the frequency of categorical variables. We will use Kaplan–Meier to analyze the TTCI (compared with a log-rank test).

**STUDY POPULATION**

**Participation**

One hundred COVID-19 confirmed cases (using the RT-PCR test or CT-scan) will be enrolled in the trial between August 20^th^ to September 4^th^, 2020. the patients will be randomly assigned in a 1:1 ratio to one of the two following therapeutic regiments; 50 were assigned to the high dose IFN-β1a group; 50 were assigned to the low dose IFNβ1a group (control group).

**Inclusion Criteria**

- Age ≥ 18
- COVID-19 Confirmed Cases by Means of RT-PCR OR CT-scan
- Oxygen saturation (SPO2) ≤ 93% OR respiratory rate ≥ 24
- At least one of the following: radiation contactless body temperature temperature ≥37.8, Cough, shortness of breath, nasal congestion/ discharge, myalgia/arthralgia, diarrhea/vomiting, headache or fatigue on admission.
- Time of onset of the symptoms should be acute (Days ≤ 14)

**Exclusion Criteria**

• Refusal to participate expressed by patient or legally authorized representative if they are present

• Patients using drugs with potential interaction with Lopinavir/Ritonavir, Interferon-β 1a

• Pregnant or lactating women.

• History of alcohol or drug addiction in the past 5 years.

• Blood ALT/AST levels > 5 times the upper limit of normal on laboratory results.

• The patients who were intubated less than one hour after admission to hospital.

**Drop Out**

In the event of developing an allergic reaction to the drug or the patient's refusal to cooperate, the participant will be considered as a dropout. Any other reason resulting in discontinuation of the therapeutic regiments would also be considered as a dropout.

**RESTRICTIONs**

Our study has several limitations. The trial will be open-label and without a placebo-control group, which opens the possibility for risks of bias. Our trial will be carried out in a limited resource setting, where we have no access to the follow-up RT-PCT testing and quantitative Real-Time RT-PCR; therefore, we could not determine the time to a negative RT-PCR test and the viral loads to shed further light on the effect of the studied drugs on viral dynamics. Finally, we will only enroll the severe patients with lower probabilities of survival; hence our findings cannot be extrapolated to all COVID-19 patients.

**ETHICAL CONCIDERATION**

• Before conducting present study, the approval of the ethics committee and the research council of Shahid Beheshti University of Medical Sciences will be obtained.

• All research steps will be recorded in IRCT.

• Written consent is obtained from all patients before entering the study.

• We will provide patients with complete and clear information about the research process.

• At each phase of the study, patients are allowed to leave the study.

• The medical record of all patients are fully preserved

**REFERENCES**

1. World Health Organization(Who). [. Available from: [https://www.who.int/emergencies/diseases/novel-coronavirus-2019?gclid=Cj0KCQjw6ar4BRDnARIsAITGzlBtMrYKhI0XLI2RFjkJM0qX4UnlfjKcHa74w49I5I1J1XM043G7d4aAhGAEALw_wcB](https://www.who.int/emergencies/diseases/novel-coronavirus-2019?gclid=Cj0KCQjw6ar4BRDnARIsAITGzlBtMrYKhI0XL-I2RFjkJM0qX4UnlfjKcHa74w49I5I1J1XM043G7d4aAhGAEALw_wcB).

2. Virological.org.Novel 2019 Coronavirus Genome. [Available from: <http://virological.org/t/issues-with-sars-cov-2-sequencingdata/>437

3. Yan R, Zhang Y, Li Y, Xia L, Guo Y, Zhou Q. Structural basis for the recognition of SARS-CoV-2 by fulllength. Science. 2020;367(6485).

4. Ou X, Liu Y, Lei X, Li P, Mi D, Ren L, et al. Characterization of spike glycoprotein of SARS-CoV-2 on virus entry and its immune cross-reactivity with SARS-CoV. Nat Commun. 2020;11(1).

5. Jose RJ, Manuel A. COVID-19 cytokine storm: the interplay between inflammation and coagulation. Lancet Respir Med. 2020;8(6):46-7.

6. Zheng H, Zhang M, Yang C-X, Zhang N, Wang C-X, Yang X-P. Elevated exhaustion levels and reduced functional diversity of T cells in peripheral blood may predict severe progression in COVID-19 patients. Cell Mol Immunol. 2020;17.

7. Wang BX, Fish EN. Global virus outbreaks: Interferons as 1st responders. SeminImmunol. 2019;43.

8. Hung IF, Lung KC, Tso EY, Liu R, Chung T, Chu M, et al. Triple combination of interferon beta-1b,lopinavir-ritonavir, and ribavirin in the treatment of patients admitted to hospital with COVID-19: an open-label,randomised, phase 2 trial. Lancet. 2020;395:1695-704.

9. Hensley LE, Fritz LE, Jahrling P, Karp CL, Huggins JW, Geisbert TW. Interferon-β 1a and SARS Coronavirus Replication. Emerg Infect Dis. 2004;10(2):317-9.

10. Farzaneh D, Nadjic SA, Saffaeid A, Marjanie M, Moniric A, Jamaatib H, et al. Subcutaneous administration of interferon beta-1a for COVID-19: A noncontrolled prospective trial. International Immunopharmacology.August 2020;85.

11. Latouche A, Porcher R, Chevret S. Sample size formula for proportional hazards modelling of competing risks. Statistics in medicine. 2004;23(21):3263-74.
